# Relation of Carotid Artery Diameter With Cardiac Geometry and Mechanics in Heart Failure With Preserved Ejection Fraction

**DOI:** 10.1161/JAHA.112.003053

**Published:** 2012-12-19

**Authors:** Zhen‐Yu Liao, Ming‐Cheng Peng, Chun‐Ho Yun, Yau‐Huei Lai, Helen L. Po, Charles Jia‐Yin Hou, Jen‐Yuan Kuo, Chung‐Lieh Hung, Yih‐Jer Wu, Bernard E. Bulwer, Hung‐I Yeh, Cheng‐Ho Tsai

**Affiliations:** 1 Division of Cardiology, Department of Internal Medicine, Mackay Memorial Hospital, Taipei, Taiwan (Z.Y.L., M.C.P., Y.H.L., C.J.Y.H., J.Y.K., C.L.H., Y.J.W., H.Y., C.H.T.); 2 Department of Radiology, Mackay Memorial Hospital, Taipei, Taiwan (C.H.Y.); 3 Department of Neurology, Mackay Memorial Hospital, Taipei, Taiwan (H.L.P.); 4 Mackay Medicine, Nursing and Management College, Taipei Medical University, Taipei, Taiwan (C.J.Y.H., C.L.H.); 5 Mackay Medical College, New Taipei County, Taiwan (J.Y.K., Y.J.W., H.Y., C.H.T.); 6 Department of Health Industry Management, Kainan University, Taoyuan, Taiwan (C.L.H.); 7 The Institute of Health Policy and Management, College of Public Health, National Taiwan University, Taipei, Taiwan (C.L.H.); 8 Department of Diagnostic Medical Imaging, School of Medical Imaging and Therapeutics, Massachusetts College of Pharmacy and Health Sciences, Boston, MA (B.E.B.); 9 Nonivasive Cardiovascular Research, Cardiovascular Division, Brigham and Women's Hospital, Boston, MA (B.E.B.)

**Keywords:** cardiac mechanics, carotid artery diameter, heart failure, hypertension, remodeling, strain

## Abstract

**Background:**

Central artery dilation and remodeling are associated with higher heart failure and cardiovascular risks. However, data regarding carotid artery diameter from hypertension to heart failure have remained elusive. We sought to investigate this issue by examining the association between carotid artery diameter and surrogates of ventricular dysfunction.

**Methods and Results:**

Two hundred thirteen consecutive patients including 49 with heart failure and preserved ejection fraction (HFpEF), 116 with hypertension, and an additional 48 healthy participants underwent comprehensive echocardiography and tissue Doppler imaging. Ultrasonography of the common carotid arteries was performed for measurement of intima‐media thickness and diameter (CCAD). Cardiac mechanics, including LV twist, were assessed by novel speckle‐tracking software. A substantial graded enlargement of CCAD was observed across all 3 groups (6.8±0.6, 7.7±0.73, and 8.7±0.95 mm for normal, hypertension, and HFpEF groups, respectively; ANOVA *P*<0.001) and correlated with serum brain natriuretic peptide level (*R*^2^=0.31, *P*<0.001). Multivariable models showed that CCAD was associated with increased LV mass, LV mass‐to‐volume ratio (β‐coefficient=10.9 and 0.11, both *P*<0.001), reduced LV longitudinal and radial strain (β‐coeffficient=0.81 and −3.1, both *P*<0.05), and twist (β‐coefficient=−0.84, *P*<0.05). CCAD set at 8.07 mm as a cut‐off had a 77.6% sensitivity, 82.3% specificity, and area under the receiver operating characteristic curves (AUROC) of 0.86 (95% CI 0.80 to 0.92) in discriminating HFpEF. In addition, CCAD superimposed on myocardial deformation significantly expanded AUROC (for longitudinal strain, from 0.84 to 0.90, *P* of ΔAUROC=0.02) in heart failure discrimination models.

**Conclusions:**

Increased carotid artery diameter is associated with worse LV geometry, higher brain natriuretic peptide level, and reduced contractile mechanics in individuals with HFpEF.

## Introduction

Heart failure with preserved ejection fraction (HFpEF) has emerged as an important public health concern. It is associated with high rates of clinical events similar to systolic heart failure and is linked to the growth of an aging population.^1^ Currently, there are ongoing efforts to identify the specific mechanisms and useful clinical markers for early‐stage myocardial dysfunction that are related to chronic hemodynamic load status, presumably a key pathological factor involved in the development of HFpEF.^2–3^ Several cardiovascular risk factors, primarily arterial hypertension (HTN) and aging, can modify the structure and function of the myocardium as well as the central arteries, a process known as cardiovascular remodeling.^4–5^ Such remodeling has been linked to altered ventricular geometry,^6–7^ the development of heart failure,^8–12^ and to worse clinical outcomes.^4,13–14^

Reduced aortic wall elasticity^15–16^ and enlarged arterial diameter^4^ in response to chronic increase in load status have been associated with vascular wall thickening—an adaptive response to wall tension increases according to the law of Laplace.^17^ However, data supporting central arterial remodeling in the transition from HTN to heart failure have not been extensively examined.

Recent advances in echocardiography techniques such as speckle‐tracking–based myocardial deformation imaging facilitate the quantitative assessment of subclinical myocardial dysfunction.^18^ In addition, such imaging techniques can provide a more comprehensive understanding of cardiac mechanics and the adaptive response in patients with various cardiovascular risks.^19^

The goal of this study was to investigate vascular remodeling and associated hemodynamics changes as exhibited in the carotid arteries, in relation to cardiac geometry, cardiac mechanics, or left ventricular (LV) twist and plasma brain natriuretic peptide (BNP) level, in subjects with HTN and HFpEF.

## Materials and Methods

### Study Population

From January 2008 to December 31, 2009, we consecutively studied 213 patients retrospectively from the cardiovascular outpatient clinic at a tertiary care hospital in North Taiwan: 116 had HTN and 49 had HFpEF. The setting was based on a routine health check‐up program with noninvasive imaging study performed. The initial object was to characterize myocardial function and some associated clinical features of high cardiovascular risk subjects. Cases of HFpEF were identified on the basis of the discharge diagnosis record adjudicated by experienced cardiologists and should have documented pulmonary edema on chest radiography at the time of their index hospitalization with LV ejection fraction >50% by echocardiographic measures. An additional 48 asymptomatic, healthy participants without previous diagnosis of any systemic disease were recruited at the time of their routine annual health check‐up. Exclusion criteria were significant valvular heart (moderate to severe) diseases, a history of pacemaker implantation, valvular surgery, ongoing atrioventricular block or episodes of frequent cardiac arrhythmias that make recording difficult, acute or overt renal dysfunction (serum creatinine ≥2.5 mg/dL), severe obstructive pulmonary disease, and a history of acute coronary syndrome in the preceding 12 months. Thorough review of the data, including baseline characteristics, medical histories, smoking and physical activity, as well as detailed physical examination results, was obtained using structured questionnaires. The imaging acquirement and quantification were carried out by the same experienced technician with no changes regarding the procedures, raters, technology, or standard of care affecting the measurements over the entire 2‐year study period. Categorization into the HTN group was confirmed by detailed chart review and defined by previous HTN diagnosis (systolic blood pressure >140 mm Hg or diastolic blood pressure >90 mm Hg by sphygmomanometer) with regular medication control yet without clinical heart failure signs or symptoms. The presence of coronary artery disease (CAD) was defined as a history of a previous myocardial infarction, history of angioplasty, or >50% luminal narrowing by coronary angiography. Biochemical values including fasting glucose level, lipid profiles, blood urea nitrogen, and creatinine, were obtained with an Hitachi 7170 Automatic Analyzer (Hitachi Corp, Hitachinaka Ibaraki, Japan) from venous blood samples, with BNP was measured by the Abbott AxSYM (Abbott Diagnostics, Abbott Park, IL) method. The data preparation and study design were retrospective and had passed the institutional board review. We did not obtain oral informed consent from all of the subjects. The local ethics committee approved the design of this study in accordance with the Declaration of Helsinki (11MMHIS143).

### Echocardiography Protocol for Assessing Ventricular Geometry and Diastolic Indices

**Figure 1.**
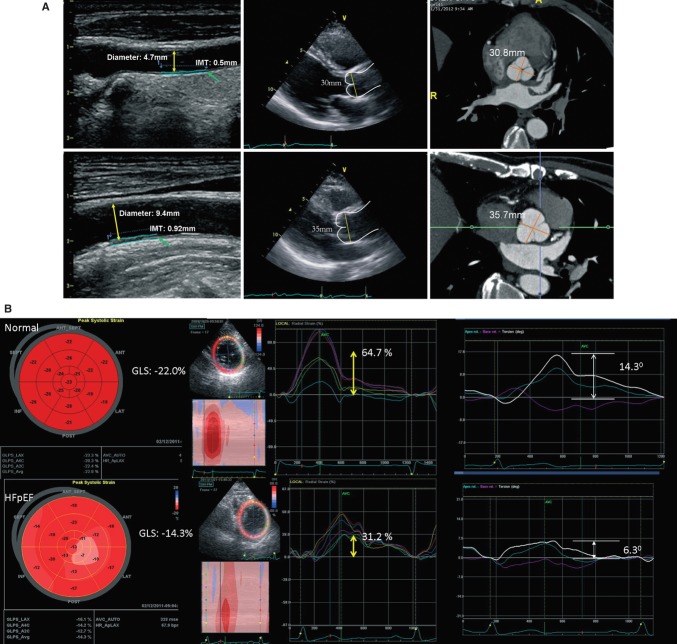
(A) The exact site and annotation for measurements of the common coronary artery diameter (CCAD) (yellow arrow; intima‐media thickness [IMT], green arrow) and aortic root diameters, and the corresponding computed tomography scanning for validation of echocardiographic measurements in healthy control subjects (top) and patients with heart failure with preserved ejection fraction (HFpEF) (bottom). (B) A bull's‐eye illustration of integrated 3‐apical views of longitudinal strain by AFI (represented as GLS: left), as well as corresponding radial strain (left ventricular [LV] mid‐wall: middle) and degree of LV twist (right). GLS indicates global longitudinal strain; AFI, automatic function imaging.

Transthoracic Doppler echocardiography examination was performed on all subjects using a commercially available ultrasound system (Vivid 7, GE‐VingMed) equipped with a 2‐ to 4‐MHz transducer (M4S). Parameters including left atrial diameter, LV internal diameter, derived LV mass, and LV mass index were determined from M‐mode measurements using American Society of Echocardiography criteria.^20–21^ The aortic root diameter was defined as the largest end‐diastolic diameter from the parasternal long‐axis view (Figure 1A, middle).

LV end‐diastolic and end‐systolic volumes were further quantified using the biplane Simpson method with ratio of LV mass to volume calculated using LV mass divided by LV end‐diastolic volume. Assessment of LV diastolic function was determined by pulsed‐wave Doppler of transmitral inflow velocities measured at the tip of the mitral leaflets with E/A ratio assessed from early (E) and late diastolic (A) filling velocities. Tissue Doppler imaging was used to determine the lateral mitral annular velocities, measuring both the peak systolic (S′) and early diastolic (E′) values. Left‐sided ventricular filling pressures (E/E′) were estimated using the ratio of the early (E) transmitral hemodynamic Doppler velocity divided by the tissue Doppler imaging–derived early diastolic (E′) lateral mitral annular velocity.

### Carotid Artery Parameters: Intima‐Media Thickness, Diameter, and Remodeling by Ultrasonography

The extracranial common carotid arteries (CCAs) were sequentially assessed using a high‐resolution 5‐ to 10‐MHz linear‐array ultrasound transducer (GE‐VingMed) by an experienced technician. All subjects had their carotid intima‐media thickness (IMT) measured at the far wall of the distal 1 cm of each CCA (Figure 1A, left). The IMT was determined by using a double‐line pattern visualized on both carotid artery walls in a longitudinal B‐mode echotomography image. CCA diameter (CCAD) was measured between the 2 leading edges of the near‐ and far‐wall intima at the same site as the IMT measurement at end‐diastole phase by ECG gating. In this study, the representative CCA IMT and CCAD were averaged from repeated 3 calculations for both the left and right CCAs. The interobserver variability (coefficient of variation) of 30 subjects randomly chosen from this study for IMT and CCAD was 4.9% and 5.6%, respectively, which had shown good reproducibility.

### Agreement of Aortic Root Diameter Measures Between Computed Tomography and Echo Methods

Thirty subjects in the study cohort (including 5 subjects from the HFpEF group, 18 with HTN, and 7 from the healthy group) had computed tomography (CT) scans (Figure 1A, right). Validation was performed for the CT‐ and echocardiography‐derived aortic root measurements. Pearson linear correlation for the echocardiography‐derived and CT‐defined aortic root diameter was 0.95 in our lab (*P*<0.001). Bland–Altman analysis of these 2 methods showed a high degree of agreement (limits of agreement: −0.217 to 4.146).

### Assessment of Myocardial Mechanics and LV Twist by Speckle‐Tracking Deformation Imaging

Baseline 2‐dimensional images (including 2‐chamber, 4‐chamber, and apical long‐axis views) for longitudinal strain and 3 short‐axis views (including mitral, papillary, and apical layer) were analyzed by offline endocardial border manual tracing, using novel offline proprietary software (version 10.8, EchoPAC, GE Vingmed Ultrasound) based on automated speckle‐tracking algorithms. The global longitudinal strain was averaged from all 3 apical views (2‐chamber, 4‐chamber, and apical long‐axis views) and displayed as a bull's‐eye map (Figure 1B, left) with global circumferential and radial strain curves obtained by averaging different values from 3 different short‐axis levels (Figure 1B, middle: mitral valve, mid‐wall, and apical levels), respectively. Cardiac twist was generated automatically by using the same sophisticated software (Figure 1B, right).^22^

### Determination of Central Arterial Pulse Pressure, Compliance, Tensile Stress, and Ventriculoarterial Function

End‐systolic pressure was estimated as systolic blood pressure×0.90 with stroke volume measured as the product of the cross‐sectional area of the LV outflow tract (measured on the parasternal long‐axis view) and the velocity‐time integral (using pulsed‐wave Doppler assessment of flow across the LV outflow tract) measured on the apical 5‐chamber view. The effective arterial elastance index was estimated as end‐systolic pressure/stroke volume with LV end‐systolic elastance calculated using the modified single‐beat measure.^5,23^ Coupled ventriculoarterial function was thus evaluated by arterial elastance index/end‐systolic elastance. The pulse pressure (PP) was determined using the brachial systolic blood pressure minus the diastolic blood pressure. The central PP was estimated by a regression equation used for the brachial PP determined by carotid applanation tonometry.^14^ Central artery compliance was estimated by calculating the ratio of stroke volume to PP:
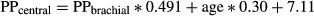
1

The wall tensile stress of the carotid artery (carotid tensile stress), during the end‐diastole phase, was estimated by the product of the mean blood pressure and one‐half of the ratio of the lumen diameter (carotid radius) to the IMT ([pressure×radius]/IMT).^24^

### Data Analysis

Continuous data are reported as the mean±SD and compared using a nonparametric trend test (Wilcoxon rank sum test) across ordered age groups with categorical or proportional incidence data expressed as a proportion and compared with use of the χ^2^ or Fisher exact test, as appropriate. One‐way ANOVA was performed to test the differences in the continuous data among the 3 groups with the post‐hoc Bonferroni correction for paired comparisons. Because the carotid IMT and remodeling depend largely on age and other cardiovascular risk factors, an ANCOVA was also used to adjust for relevant covariates in the comparison of mean values among the 3 groups. The minimum required samples needed to detect significant difference between the HFpEF and HTN groups by longitudinal myocardial deformation (strain), a sensitive and powerful clinical marker for such purpose,^25^ was calculated. A reported mean of −14.9 and SD of 2.9 in the HFpEF^25^ group and mean of −16.9 and SD of 4.1 in the HTN^26^ group resulted in an effect size (Cohen's *d*) of 0.563. Given that the subjects with HFpEF had a smaller amount with a potentially wide range of longitudinal strain value compared with subjects with HTN (from −15.9% to −18.8%),^26^ the authors prespecified the ratio as a 2:1 allocation ratio, leading to a sample size of nearly 100:50 for HTN and HFpEF, respectively, with an power of 90% and an α rate of 5%. The sample size calculation was performed by using G*Power 3.1.5 (University Kiel, kiel, Germany). Additionally, a normal control group was included to contrast the difference from the HTN and HFpEF groups.

The diagnostic and incremental values of the CCAD superimposed on diastolic parameters and myocardial deformation data were assessed using receiver operating characteristic (ROC) curves with the optimal cut‐off chosen for maximum sensitivity and specificity for the clinical diagnosis of HFpEF. The incremental value between models was assessed by changes of area under ROC (ΔAUROC), with *P* value provided for statistical significance (*P* of ΔAUROC). To avoid overspecification, calibration with 5‐fold cross‐validation was performed. A multivariable regression model was used to determine the significance of the covariate‐adjusted relation between myocardial deformation data and clinical variables, biochemical profiles and echo‐derived measurements of the aortic root, and carotid artery diameters with individual odds ratios, and *P* values and 95% CIs.

All data were analyzed with use of the STATA 9.0 software package (StataCorp). The *P* value was set for 2‐tailed probability with a *P* value <0.05 considered statistically significant.

## Results

### Baseline Demographic Data

The baseline demographic data for the 213 participants enrolled in this study are presented in Table 1. Compared with the control and HTN groups, subjects with HFpEF were more likely to be female (67.4%), and have a larger body mass index (all *P*<0.05). Both the HTN and HFpEF groups had higher systolic and diastolic blood pressure (all *P*<0.05). Moreover, both the HTN and HFpEF groups had higher blood sugar levels, worse renal function, lower serum HDL and Hb, and higher serum high‐sensitivity C‐reactive protein and BNP levels than the control group (all *P*<0.05).

**Table 1.** Baseline Demographics and Laboratory Data for the Study Cohort

	Healthy Group (n=48)	HTN Group (n=116)	HFpEF Group (n=49)	Trend *P* Value
Baseline demographics				
Age, y	50.7±10.1	62.9±13.9*	72.5±11*†	<0.001
Sex, female	17 (35.4%)	46 (40%)	33 (67.4%)	0.002
Height, cm	166±7.9	162.4±9.9*	157±7.7*†	0.0007
Weight, kg	65.2±11.7	66.5±13.7	69.4±11.3	0.17
Body mass index, kg/m^2^	23.6±3.3	25.2±3.8*	28.1±3.1*†	<0.001
Systolic blood pressure, mm Hg	118.02±13.2	142.6±18.9*	145±20.9*	<0.001
Diastolic blood pressure, mm Hg	70±8	88±15.6*	80±13.5†	0.005
Central pulse pressure, mm Hg	45.8±5.9	53±9.2*	60.7±9.8*†	<0.001
History of hypertension, %	—	116 (100)	39 (80)	<0.001
History of diabetes, %	—	40 (34.5)	21 (42.9)	<0.001
History of CAD, %	—	28 (24.1)	20 (40.8)	<0.001
Laboratory data				
Fasting glucose, mg/dL	98.2±10.4	116.3±30.4*	133.3±41.5*†	0.005
Cholesterol, mg/dL	197.9±35.1	187.8±39.3	183.3±44.5	0.04
Triglycerides, mg/dL	116.5±64.7	136±68.9	138.5±73.5	0.01
Low‐density lipoprotein, mg/dL	107.7±29.5	141.9±40.8	141.5±57.7	0.711
High‐density lipoprotein, mg/dL	57.6±15.7	47.9±14.8*	44.9±13.9*	0.005
eGFR, mL/min/1.73 m^2^	87.3±13.1	79.2±25.4	62.7±29.2*†	0.005
Hemoglobin, g/dL	14.8±1.3	13.9±1.8*	13.1±2.2*†	0.0017
C‐reactive protein, mg/dL (mean, 25th to 75th)*	0.15 (0.1–0.18)	0.32 (0.12–0.46)	1.67 (0.17–1.8)*†	0.0195
BNP, pg/mL (mean, 25th to 75th)‡	28.4 (6.9–39.9)	36 (9.75–48.5)	444.9 (87.6–594)*†	<0.001

BNP indicates brain natriuretic peptide; CAD, coronary artery disease, eGFR, estimated glomerular filtration rate; HFpEF, heart failure with preserved ejection fraction; HTN, hypertension.

ANOVA Bonferroni post hoc test:

* *P* value <0.05 vs healthy group;

† *P* value <0.05 vs HTN group.

‡ The differences were compared by log values.

### Ventricular Geometry, Diastolic Indices, Central Hemodynamics, and Ventriculoarterial Function

Table 2 shows the comparisons of the baseline conventional echocardiographic measurements among the 3 groups. Compared with the control group, patients with HTN and HFpEF tended to have higher LV wall thickness, higher transmitral A‐wave velocities, prolonged transmitral E‐wave deceleration time and isovolumic relaxation time, higher LV wall stress, and significantly reduced mitral annular relaxation velocity E′ (all *P*<0.05). Both vascular elastance (arterial elastance index) and LV end‐systolic elastance reflecting vascular and ventricular stiffness were worse in the HFpEF group (*P*<0.05), whereas ventriculoarterial coupling (arterial elastance index/end‐systolic elastance) remained similar across 3 groups. Furthermore, the patients with HFpEF also had a significantly higher aortic root and left atrial diameter; greater wall thickness, LV mass index, relative wall thickness, and mass‐to‐volume ratio; and reduced stress‐corrected mid‐wall function (all *P*<0.05). In addition, both the HTN and HFpEF groups had higher estimated central pulse pressures compared with the healthy group (both *P*<0.05). A significantly higher left‐sided ventricular filling pressure, by E/E′, was also observed in the HFpEF group (*P*<0.05).

**Table 2.** Differences in Baseline Ventricular Geometry, Diastolic Indices, Ventriculoarterial Function, and Speckle‐Tracking–Based Myocardial Mechanics and LV Twist Among the 3 Groups

LV Structures and Function	Healthy Group (n=48)	HTN Group (n=116)	HFpEF Group (n=49)	Trend *P* Value
Conventional echocardiographic measures				
Aortic root diameter, mm	32.2±3.7	34.4±3.8*	35.9±4.5*	<0.001
LV ejection fraction, %	66.6±6.7	68.9±7.4	68.6±7.5	0.2
Relative wall thickness	0.39±0.06	0.48±0.07*	0.55±0.09*†	<0.001
Left atrial diameter, mm	31.5±4.8	33.1±5	35.8±5.7**	<0.001
LV mass, g	128.7±29.4	162.8±41.6*	193±58.6*†	<0.001
LV mass index, gm/m^2^	69.4±14.4	88±21.9*	103.5±30.2*†	<0.001
LV M/V ratio	1.4±0.2	1.8±0.3*	2.1±0.4*†	<0.001
Fscmw, %	23.5±2.9	21.8±2.9*	20.7±2.9*	<0.001
cESS, kdyne/cm^2^	75.6±15.2	93±26.9*	88.7±28.6*	0.03
Doppler echocardiographic study				
Mitral E DT, ms	194.5±40.7	240±46*	245.9±73†	0.005
IVRT, ms	83.2±10.5	102.1±18.8*	86.7±18.4†	0.55
Lateral E′, cm/s	10.4±2.4	7.5±2.1*	5.9±1.5*†	<0.001
Lateral S′, cm/s	10±3	7.8±2.3*	6.5±1.9*†	<0.001
Lateral E/E′, mmHg	6.9±1.7	9.4±4.2	14.6±6.9*†	<0.001
Ventriculoarterial function				
Ea, mm Hg/mL	1.88±0.51	2.12±0.64	2.19±0.66*	0.01
Ees, mm Hg/mL	2.8±0.9	3.3±1.3	3.4±1.4*	0.03
Ea/Ees	0.71±0.21	0.71±0.26	0.72±0.25	0.93
Myocardial mechanics				
Longitudinal S, %	−19.9±2.0	−17.8±1.8*	−13.9±2.9*†	<0.001
Radial S, %	45.4±10.3	37.1±11.7*	26.2±10.4*†	<0.001
Circumferential S, %	−21.2±2.9	−21.2±3.5	−18.7±5.3*	0.005
Twist, °	13.3±3.4	13.2±3.7	11.1±4.4*†	0.005

LV indicates left ventricular; HTN, hypertension; HFpEF, heart failure with preserved ejection fraction; M/V ratio, mass‐to‐volume ratio; Fscmw, stress‐corrected mid‐wall fractional shortening; cESS, ventricular circumferential wall stress; DT, transmitral E‐wave deceleration time; IVRT, isovolumic relaxation time; E′, early mitral annular relaxation velocity; S, mitral annulus systolic velocity; Ea, vascular elastance; Ees, LV end‐systolic elastance; S, strain.

ANOVA Bonferroni post hoc test:

* *P* value <0.05 vs healthy group;

*P* value <0.05 vs HTN group.

Myocardial deformation, from both longitudinal and radial measures, was significantly lower in both the HTN and HFpEF groups compared with the normal controls (all *P*<0.05). In addition, LV twist behavior was significantly lower in the HFpEF group (*P*<0.05).

### Carotid Artery IMT, Diameter, and Remodeling and Association with Central Hemodynamics

**Figure 2.**
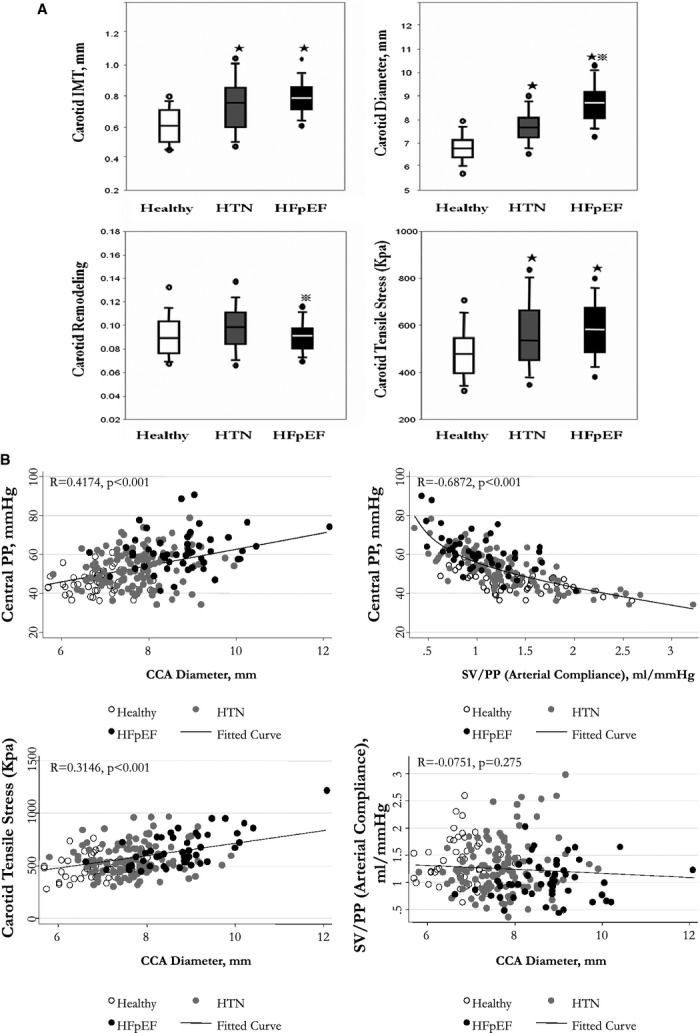
(A) Individual central artery intima‐media thickness (IMT), diameters, and remodeling. Both the hypertension (HTN) and heart failure with preserved ejection fraction (HFpEF) groups had higher IMT than the control group. Patients with HFpEF had significantly greater carotid artery diameters compared with both the control and HTN groups. The common carotid artery (CCA) remodeling (IMT‐to‐diameter ratio) was largest in the HTN group, indicating that the increase in the CCA diameter was greater than the increased IMT. The central artery compliance, ventriculoarterial coupling, and wall stress for both the carotid artery and left ventricular wall are shown (B, top). The fitting curve or linear regression used for comparison of the central pulse pressure (PP) and CCA diameter (left) and, central PP and arterial compliance, among study patients is shown (A, bottom). Compared with the healthy control and HTN groups, subjects with HFpEF had larger central artery pressure related to larger carotid artery diameter and poorer arterial compliance. SV indicates stroke volume.

Figure 2A shows that both the HTN and HFpEF groups had larger carotid artery IMT, higher carotid artery tensile stress (both *P*<0.05 compared with the healthy group), and a graded substantial enlargement of CCAD from the healthy, HTN, to HFpEF groups (6.8±0.6, 7.7±0.73, and 8.7±0.95, respectively; ANOVA *P*<0.001) even when adjusted for age, body mass index, and blood pressure. A significantly decreased carotid artery IMT‐to‐diameter ratio (carotid artery remodeling) was also observed in HFpEF compared with the HTN group (ANOVA *P*<0.001). Figure 2B shows the association between the CCAD and central artery hemodynamics. Larger CCAD was associated with higher central artery pulse pressure (*r*=0.42, *P*<0.001) and elevated carotid artery tensile stress (*r*=0.31, *P*<0.001); however, there was no direct relationship between the arterial compliance and CCAD (*r*=−0.075, *P*=0.275).

### Association Among Carotid Artery Parameters, Myocardial Mechanics, and LV Twist

The association among ventricular geometry, aortic diameter, and parameters of carotid arteries was shown in Table 3. Both enlargement aortic and carotid artery diameters were associated with larger LV mass and mass‐to‐volume ratio, either univariable or multivariable models (all *P*<0.05). In addition, there was an association between larger aortic diameter and a reduction in longitudinal and radial strain (β‐coefficient=1.26 and −3.72, respectively; both *P*<0.05) or twist (β‐coefficient=−1.07, *P*<0.05), although these associations were less pronounced in the multivariable models (Table 4). In addition, both larger CCA IMT and CCAD were significantly associated with worse myocardial deformation parameters (all *P*<0.05) in the univariable model. In the multivariable models, larger CCAD remained significantly independent of reduced longitudinal, radial strain, and degree of LV twist (β‐coefficient=0.81, −3.1 and −0.84, respectively; all *P*<0.05).

**Table 3.** Association Among Central Artery Diameter, Remodeling, and LV Geometry in Terms of LV Mass and LV Mass‐to‐Volume (LV M/V) Ratio

Models Used	LV Mass (g)	LV M/V Ratio
	β‐Coefficient (95% CI)	β‐Coefficient (95% CI)
Model 1		
Aortic root diameter	26.5 (20.8–32.3)*	0.16 (0.10–0.22)*
CCA IMT	12 (5.5–18.5)*	0.14 (0.09–0.2)*
CCAD	24 (18.3–29.8)*	0.22 (0.17–0.27)*
CCA IMT‐to‐lumen ratio	—	—
Model 2		
Aortic root diameter	19.4 (12.8–26)*	0.12 (0.06–0.18)*
CCA IMT	8.4 (1.8 to 15)*	0.06 (0.008–12)*
CCAD	17.5 (10.7–24.3)*	0.13 (0.07–0.19)*
CCA IMT‐to‐lumen ratio	—	—
Model 3		
Aortic root diameter	13.1 (6.4–19.8)*	0.07 (0.009–0.14)*
CCA IMT	—	—
CCAD	10.9 (3.01–18.8)*	0.11 (0.03–0.18)*
CCA IMT‐to‐lumen ratio	—	—

LV indicates left ventricular; M/V ratio, mass‐to‐volume ratio; CCA indicates common carotid artery; IMT, intima‐media thickness; CCAD, CCA diameter.

Model 1: unadjusted model.

Model 2: adjusted for age, sex, and body mass index.

Model 3: adjusted for age, sex, body mass index, systolic blood pressure, fasting glucose, triglycerides, high‐density lipoprotein, estimated glomerular filtration rate, hypertension, diabetes, coronary artery disease, and smoking.

* *P* value <0.05.

**Table 4.** Association Among Central Artery Diameter, Remodeling, Myocardial Mechanics, and LV Twist

	β‐Coefficient (95% CI)			
Myocardial Deformation	Aortic Root Diameter	CCA IMT	CCAD	CCA IMT‐to‐Lumen Ratio
Model 1				
Longitudinal S	1.26 (0.89 to 1.64)	1.09 (0.71 to 1.47)	1.87 (1.56 to 2.18)	—
Radial S	−3.72 (−5.46 to −1.99)	−4.1 (−5.79 to −2.42)	−6.57 (−8.06 to −5.08)	—
Circumferential S	—	1.81 (0.98 to 2.63)	0.91 (0.07 to 1.75)	1.5 (0.53 to 2.48)
Twist	−1.07 (−1.59 to −0.54)	—	−1.01 (−1.51 to −0.5)	—
Model 2				
Longitudinal S	0.91 (0.54 to 1.27)	0.36 (0.06 to 0.72)	1.18 (0.83 to 1.53)	—
Radial S	−3.07 (−4.99 to −1.16)	−2.21 (−4.01 to −0.42)	−5.27 (−7.1 to −3.4)	—
Circumferential S	—	1.61 (0.68 to 2.53)	—	1.23 (0.23 to 2.24)
Twist	−0.91 (−1.55 to −0.28)	—	−0.86 (−1.49 to −0.23)	—
Model 3				
Longitudinal S	0.52 (0.11 to 0.92)	—	0.81 (0.35 to 1.26)	—
Radial S	—	—	−3.1 (−5.68 to −0.51)	—
Circumferential S	—	—	—	—
Twist	—	—	−0.84 (−1.66 to −0.03)	—

CCA indicates common carotid artery; CCAD, CCA diameter; IMT, intima‐media thickness; S, strain.

Model 1: unadjusted model.

Model 2: adjusted for age, sex, and body mass index.

Model 3: adjusted for age, sex, body mass index, systolic blood pressure, fasting glucose, triglycerides, high‐density lipoprotein, estimated glomerular filtration rate, hypertension, diabetes, coronary artery disease, and smoking.

*P value <0.05.

### Diagnostic Value of Carotid Artery Diameter in Patients With HFpEF and Correlation with Serum BNP Levels

**Figure 3.**
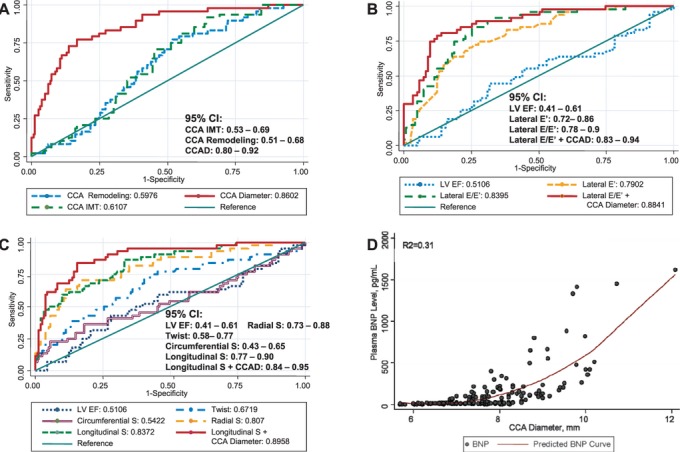
The area under the receiver operating characteristic curves used for diagnosing heart failure with preserved ejection fraction (HFpEF), based on 3 different modalities. A, Carotid artery intima‐media thickness (IMT), diameter, and remodeling were used. B, Left ventricular ejection fraction (LV EF) and diastolic measurements, including the lateral mitral annulus early diastolic velocity (E′), early mitral inflow velocity (E), and their ratio (E/E′), as well as carotid artery diameter superimposed on E/E′, are shown. C, Myocardial mechanics including longitudinal, radial, and circumferential strain (S) and LV twist are shown, as well with carotid artery diameter superimposed on longitudinal strain. The scatterplot between serum brain natriuretic peptide (BNP) level and common carotid artery diameter (CCAD) was further illustrated (D). PP indicates pulse pressure; SV, stroke volume.

**Figure 4.**
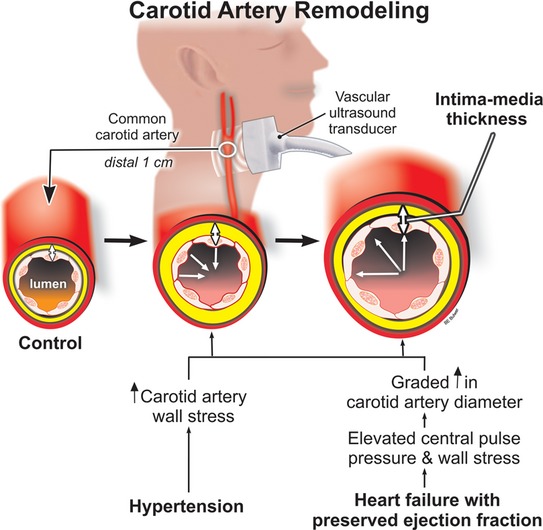
A schematic example of how carotid arterial diameter may change and remodeled in response to chronically elevated pressure load in the transition from hypertension to the development of heart failure is shown. The failure of arterial wall constraint against chronic, elevated high transmural pressure may result in progressively enlarged lumen diameter out of proportion to the compensatory increase of intima‐media thickness and, may ultimately lead to dilated carotid artery.

Figure 3 shows the diagnostic value of the CCAD, IMT, and remodeling (Figure 3A), as well as all individual echocardiography‐derived parameters including diastolic function (Figure 3B) and all myocardial deformations and twist (Figure 3C) in patients with HFpEF. In addition, the AUCs with CCAD superimposed on most commonly used clinical diastolic (E/E′) and myocardial deformation (longitudinal strain) for discriminating HFpEF were also displayed. The scatterplot between serum BNP level and CCAD was further shown in Figure 3D, suggesting that higher CCAD was associated with higher serum BNP levels (*R*^2^=0.31, *P*<0.001). As assessed with the ROC analysis, the AUROC for the diagnosis of HFpEF by using carotid artery IMT, remodeling, and CCAD was 0.61 (95% CI 0.53 to 0.69), 0.6 (95% CI 0.51 to 0.68), and 0.86 (95% CI 0.80 to 0.92), respectively. The optimal cut‐off value for carotid artery diameter, in the diagnosis of HFpEF, was 8.07 mm, which yields a sensitivity of 77.6% and a specificity of 82.3%. When CCAD was superimposed on the E/E′, the AUROC increased from 0.84 to 0.88 (95% CI 0.83 to 0.94; *P* of ΔAUROC=0.12, Figure 3B). Furthermore, when the CCAD was added into myocardial deformation imaging data, the AUROC increased significantly from 0.84 to 0.90 (for longitudinal strain, 95% CI 0.84 to 0.95; *P* of ΔAUROC=0.02, Figure 3C). Five‐fold cross‐validation showed AUROC of 0.8927 (Figure 4), with a *P* value 0.36, indicating that the model was not significantly miscalibrated.

## Discussion

The results of this study showed that the HTN and HFpEF groups had similar elevation of the LV wall stress but with graded enlargement of carotid artery diameter that tracked with higher BNP level, higher carotid artery tensile stress, and estimated central pulse pressure in a continuum from HTN to HFpEF. In addition, our result showed that an enlargement in carotid artery diameter was associated with worsening LV geometry, reduced myocardial deformation parameters, and LV twist. The addition of carotid artery diameter to the myocardial deformation parameters added incremental value for the clinical diagnosis of HFpEF.

Compensatory enlargement of the central arterial diameter (as for the CCA) in response to chronic, elevated arterial pressure was shown to contribute to rupture of the load‐bearing elastin fibers in response to mechanical fatigue resulting from higher tensile wall stress.^27–28^ Arterial remodeling, in terms of larger carotid IMT‐to‐lumen ratio, can result in negative carotid artery remodeling through proteoglycan/collage matrix deposition and smooth muscle proliferation in the media layer, in an attempt to counteract the increased tensile wall stress due to higher transmural pressures in chronic HTN.^29–33^

**Figure 5.**
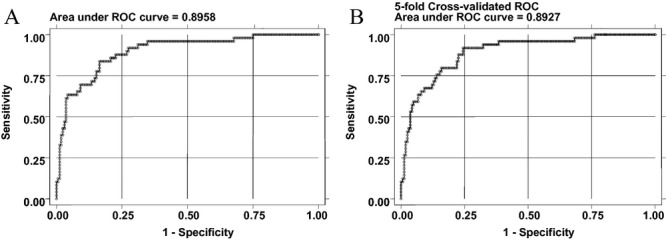
(A) The observed AUROC for HFpEF in the original model using longitudinal myocardial deformation (strain) plus CCAD=0.8958. (B) By using 5‐fold cross‐validation, the cross‐validated ROC curve is similar in shape and area under ROC (0.8927) to the one obtained in (A), with goodness‐of‐fit *P*=0.36.

Results from a previous study showed that pulse pressure–related cyclic stretch can lead to increased mechanical stress, resulting in dilatation of the large, elastic arteries (eg, CCA).^28,30^ However, such effects were less likely in the more peripheral muscular arteries.^28,34–35^ Similarly, owing to the large amount of circular and longitudinal muscular layers encircling the media layer of the aorta,^34–35^ its adaptive behavior in response to increased load may be different from that of the medium‐sized CCAs, where elastic fibers predominate.^4,36^ However, to what extent such carotid artery may remodel in the transition from HTN to HFpEF remains largely unexplored. We therefore speculated that when vascular remodeling fails to overcome the chronic, elevated mechanical stress due to pulsatile stretch and reaches a point of “no‐return,” the consequence will include the manifested dilatation of the carotid arterial lumen that may accompany the development of HF (Figure 5). Furthermore, the relatively higher carotid artery diameter and accompanied central pulse pressure widening or vascular stiffness in such remodeling process may help to explain the observed postural hypotension under conditions of volume depletion, especially in elderly patients with high‐risk profiles for HF.

Impaired endocardial longitudinal function has been shown to be a sensitive marker for identifying early‐stage diseased myocardium, particularly in patients with hypertrophy and ischemia, which may be exacerbated by elevated mechanical stress, which could be readily identified by deformation imaging.^37–40^ Because aging and HTN can play a major role in carotid artery dilatation and remodeling,^28^ it is worth emphasizing that such observed large artery remodeling in terms of either wall thickening through proteoglycan/collage matrix deposition or increased internal diameter per se may share a similar pathophysiology with ventricular remodeling and worsened myocardial deformation, a putative precursor in the development of heart failure.^6–7,41^ Indeed, we demonstrated that both remodeling patterns were in line with ventricular geometric alterations and worse ventricular contractile function in terms of lower deformation values, with larger artery diameter being more consistent even after adjusting for blood pressure and clinical variables. Unlike the triggered released of BNP^42^ as a more linear function reflecting the degree of myocardial damage from HTN to HF development, the clinical interpretation by using simply judging LV morphology for such purpose may be complicated by its phenotype diversity and the lack of consensus. Therefore, we proposed that enlarged carotid artery diameter, which tracks with increased ventriculoarterial stiffness, elevated serum BNP level, and reduction of myocardial deformation indices, could be a useful clinical marker in early identifying ventricular functional decline.

The limitations of this study include the following. First, the findings in this study are from a cross‐sectional study design and are based on a relatively small sample size with no longitudinal follow‐up data. The subjects in this study with HFpEF were older, with a higher percentage of men in the healthy group. However, there was considerable overlap with the patients with HTN of similar age, and this would not be expected to influence the results. Also, the data on the central pulse pressure were obtained from regression equations and not through invasive procedures. Furthermore, the effect of interventions on the arterial remodeling and diameter was not tested in this study, and the potential benefit of intervention in clinical practice was not investigated and should require further study.

## Conclusions

The results of this study demonstrated that larger carotid artery diameter, worse LV geometry, and impaired ventricular systolic function may progress in parallel, indicating that both large arteries and ventricles in subjects with HFpEF are prone to mechanical failure from increased load and elevated tensile stress. Therefore, enlarged carotid artery diameter may serve as useful clinical marker for identifying subjects at risk for HF.
